# Eukaryotic Initiation Factor 5B (eIF5B) Cooperates with eIF1A and eIF5 to Facilitate uORF2-Mediated Repression of ATF4 Translation

**DOI:** 10.3390/ijms19124032

**Published:** 2018-12-13

**Authors:** Joseph A. Ross, Kamiko R. Bressler, Nehal Thakor

**Affiliations:** 1 Department of Chemistry and Biochemistry, University of Lethbridge, 4401 University Drive W, Lethbridge, AB T1K 3M4, Canada; joseph.ross@uleth.ca (J.A.R.); kamiko.bressler@uleth.ca (K.R.B.); 2 Canadian Centre for Behavioral Neuroscience (CCBN), Department of Neuroscience, University of Lethbridge, 4401 University Drive W, Lethbridge, AB T1K 3M4, Canada; 3 Arnie Charbonneau Cancer Institute, Cumming School of Medicine, University of Calgary, 3280 Hospital Drive NW, Calgary, AB T2N 4Z6, Canada

**Keywords:** eukaryotic initiation factor 5B (eIF5B), eIF1A, eIF2A, upstream open reading frames (uORFs), Activating Transcription Factor 4 (ATF4), eukaryotic initiation factor 2α (eIF2α)

## Abstract

A variety of cellular stresses lead to global translation attenuation due to phosphorylation of the alpha subunit of eukaryotic initiation factor 2 (eIF2), which decreases the availability of the eIF2-GTP-Met-tRNA_i_ ternary complex. However, a subset of mRNAs continues to be translated by non-canonical mechanisms under these conditions. In fact, although translation initiation of activating transcription factor 4 (ATF4) is normally repressed by an upstream open reading frame (uORF), a decreased availability of ternary complex leads to increased translation of the main ATF4-coding ORF. We show here that siRNA-mediated depletion of eIF5B—which can substitute for eIF2 in delivering Met-tRNA_i_—leads to increased levels of ATF4 protein in mammalian cells. This de-repression is not due to phosphorylation of eIF2α under conditions of eIF5B depletion. Although eIF5B depletion leads to a modest increase in the steady-state levels of *ATF4* mRNA, we show by polysome profiling that the depletion of eIF5B enhances ATF4 expression primarily at the level of translation. Moreover, eIF5B silencing increases the expression of an ATF4-luciferase translational reporter by a mechanism requiring the repressive uORF2. Further experiments suggest that eIF5B cooperates with eIF1A and eIF5, but not eIF2A, to facilitate the uORF2-mediated repression of ATF4 translation.

## 1. Introduction

Translation of mRNA is critical yet highly energy-intensive, necessitating its stringent regulation [1]. Moreover, dysregulation of translation causes pathophysiological disorders, such as cancer [2]. Eukaryotic translation is regulated primarily at the initiation stage, involving more than a dozen eukaryotic initiation factors (eIFs) [3]. Physiological stress conditions lead to modifications of key eIFs, attenuating global mRNA translation. For example, phosphorylation of eIF2α is a well-characterized mechanism for preventing the translation of most mRNAs. However, non-canonical translation initiation mechanisms allow for the selective translation of a subset of mRNAs under such conditions [1,3,4].

eIF2 is required to form the “ternary complex”, which delivers the initiator Met-tRNA_i_ to the 40S ribosomal subunit and is essential for translation initiation [3]. eIF2 exists in either a GDP- or GTP-bound state. Hydrolysis of eIF2-bound GTP is required for the transfer of Met-tRNA_i_ to the 40S ribosomal subunit, releasing GDP. The exchange of GDP for GTP is catalyzed by the guanine exchange factor, eIF2B, and is necessary for the regeneration of active ternary complex [3]. In response to a wide variety of stresses, such as viral infection, osmotic shock, or hypoxia, the alpha subunit of eIF2 is phosphorylated at serine 51, increasing its binding affinity for eIF2B and sequestering both proteins in an inactive complex (reviewed in [5]). The cellular concentration of eIF2B is limiting, such that even a low proportion of eIF2α phosphorylation results in inhibition of ternary complex re-formation [6]. Consequently, translation initiation is attenuated for most mRNAs. There are four kinases that act to phosphorylate eIF2α in response to stress: haem-regulated inhibitor (HRI), protein kinase activated by double-stranded RNA (PKR), general control non-derepressible-2 (GCN2), and PKR-like endoplasmic reticulum kinase (PERK); collectively, the down-regulation of global translation mediated by these proteins and eIF2 is known as the integrated stress response (ISR) (reviewed in reference [5]).

Although global translation is inhibited during stress conditions, the translation of many mRNAs is unaffected by phosphorylation of eIF2α. In fact, the translation of some mRNAs is increased under conditions of eIF2α phosphorylation, such as activating transcription factor 4 (ATF4). The *ATF4* mRNA encodes two short upstream open reading frames (uORFs) in its 5′ untranslated region (5′ UTR). uORFs are mRNA elements in the 5′ UTR of a protein-coding gene with a start codon that is out of frame with the main coding sequence [7]. As ribosomes load onto the 5′ cap of mRNA transcripts and scan for the first start codon, uORFs typically disrupt the translation of the downstream coding sequence. In the case of the ATF4 transcript, the 5′-most uORF (uORF1) encodes just 3 codons, while uORF2 encodes 59 codons and overlaps the start codon of the main ATF4 ORF [8]. Under normal conditions—when the ternary complex is relatively abundant—these uORFs engage the ribosome and initiation at uORF2 prevents initiation at the main coding sequence [9], resulting in low levels of ATF4 translation initiation (reviewed in reference [5]). However, during stress conditions, the availability of ternary complex becomes limited, which increases the probability that ribosomes will skip uORF2 without initiating. Therefore, when ternary complex concentration is low, more ribosomes will bypass uORF2 and initiate translation of the main coding sequence [9].

ATF4 regulates the transcription of many stress-response genes and is a master regulator of cellular adaptation to stress [10]. ATF4 binds to C/EBP-ATF response element (CARE) sequences of its target genes, including C/EBP homologous protein (CHOP), which is also a transcription factor that increases expression of a set of stress-response genes [10,11]. Another downstream target of ATF4 is growth arrest and DNA damage-inducible protein 34 (GADD34), which acts as a point of negative feedback in the ISR: when activated, GADD34 binds and activates protein phosphatase 1 (PP1), thus reversing the phosphorylation of eIF2α and inactivating the ISR [11,12].

A recent body of evidence suggests that another initiation factor, eIF5B, is able to substitute for eIF2 functionality in at least some contexts. For instance, under standard growth conditions, X-linked inhibitor of apoptosis (XIAP) is produced via canonical eIF2-dependent translation initiation. However, under conditions of cellular stress and eIF2α phosphorylation, IRES-dependent translation of XIAP mRNA relies on eIF5B [13]. eIF5B is homologous to bacterial and archaeal IF2, which delivers Met-tRNA^fMet^ to bacterial/archaeal ribosomes [14,15]. Under standard conditions, eIF5B is responsible for assisting in the joining of the 40S and 60S ribosomal subunits, as well as playing a role in stabilizing Met-tRNA_i_ binding [16]. eIF5B was also shown to deliver Met-tRNA_i_ into the P-site of the ribosome in an eIF2-independent translation initiation mechanism utilized by the CSFV and HCV IRESs [17,18,19]. Thus, eIF5B appears to be capable of substituting for eIF2 in Met-tRNA_i_-delivery to the ribosome. Additionally, eIF5B was shown to act as an essential translation factor during hypoxia by facilitating Met-tRNA_i_ delivery to ribosomes for efficient cap-dependent translation of hypoxia-response proteins in glioblastoma cells [20]. We have recently demonstrated a role for eIF5B in the non-canonical translation of several anti-apoptotic and pro-survival proteins involved in glioblastoma progression and resistance to therapeutic agents [21]. In yeast cells, eIF5B has been shown to regulate translation of upstream open reading frame (uORF)-containing mRNAs involved in amino acid biosynthesis [22]. In mammalian cells, eIF5B has been shown to regulate cell cycle progression via regulating uORF-containing mRNAs such as p27 and p21 [6].

These findings suggest a role for eIF5B in non-canonical mechanisms of translation initiation under cellular stress conditions. As eIF5B can apparently substitute for eIF2α in delivering Met-tRNA_i_ during translation initiation [17,18,19,20], we hypothesized that eIF5B might play a role in the uORF-mediated regulation of ATF4 translation. We show here that depletion of eIF5B by RNAi leads to increased levels of ATF4 protein in two cell lines (HEK293T and U20S), which is not due to a general phosphorylation of eIF2α under conditions of eIF5B depletion. Depletion of eIF5B also leads to increased mRNA and protein levels of a downstream member of the ATF4 regulon, GADD34. Although eIF5B depletion leads to a modest increase in the steady-state levels of *ATF4* mRNA, a robust increase in ATF4 translation is observed by polysome profiling analysis, suggesting that eIF5B represses ATF4 expression primarily at the level of translation. Moreover, eIF5B depletion leads to increased expression of an *ATF4*-luciferase translational reporter, and this de-repression requires intact uORF2. Finally, depletion of eIF1A or eIF5 causes increased expression of ATF4, which is not synergistic with that caused by eIF5B depletion, suggesting that eIF5B cooperates with each of these factors in order to repress ATF4 translation. Together, our data suggest that eIF5B facilitates the uORF2-mediated repression of ATF4 translation.

## 2. Results

### 2.1. eIF5B Represses ATF4 Independently of eIF2α

**Figure 1:**
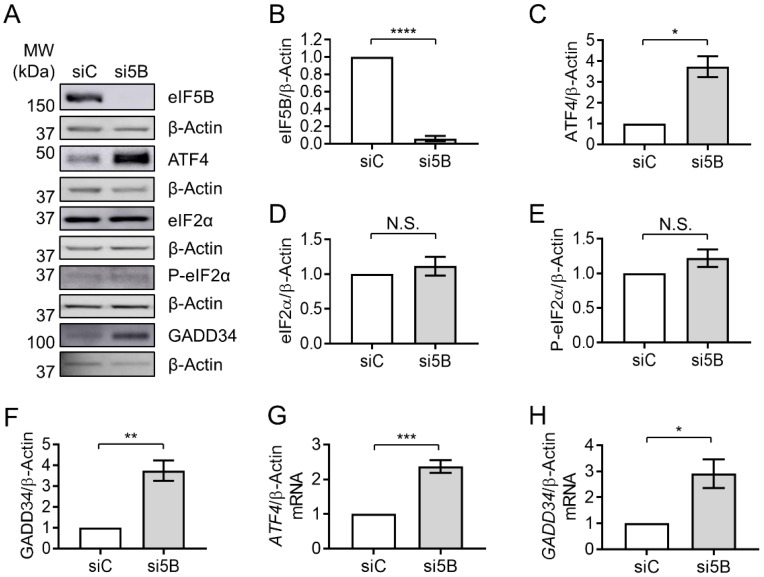
Depletion of eIF5B leads to increased levels of the ATF4 protein. HEK293T cells were reverse-transfected with a non-specific control siRNA (siC) or an eIF5B-specific siRNA pool (si5B), incubated 96 h, harvested in RIPA lysis buffer, and 20 µg of total protein resolved by SDS-PAGE before performing immunoblotting. (**A**) Representative images of immunoblots probing for eIF5B, ATF4, eIF2α, P-eIF2α, GADD34, or β-Actin (internal control). (**B**–**H**) Quantitation of eIF5B (**B**), ATF4 (**C**), eIF2α (**D**), P-eIF2α (**E**), or GADD34 (**F**), normalized to β-Actin, from HEK293T cells. (**G**,**H**) Total RNA was isolated from control or eIF5B-depleted HEK263T cells and subjected to RT-qPCR analysis of steady-state mRNA levels for ATF4 (**G**) or GADD34 (**H**), normalized to β-Actin mRNA. Data are expressed as mean ± SEM for at least 3 (**B**–**F**) and up to 4 (**G**,**H**) independent biological replicates. * *p* < 0.05; ** *p* < 0.01; *** *p* < 0.001; **** *p* < 0.0001. N.S., not statistically significant.

Depletion of eIF5B led to a significant increase in ATF4 proteins levels in HEK293T cells (~3.7-fold; Figure 1A–C), suggesting a repressive role for eIF5B in ATF4 expression. A similar increase in ATF4 levels was observed in U20S cells upon eIF5B depletion (~5-fold; Figure S1A–C), suggesting that this repressive role is not limited to the HEK293T cell line. As ATF4 levels are known to be up-regulated by eIF2α-phosphorylation, we determined whether eIF5B depletion might be indirectly enhancing ATF4 levels through a general stress-mediated phosphorylation of eIF2α. Levels of total and phospho-eIF2α were unchanged upon eIF5B depletion in HEK293T (Figure 1A,D,E). In U2OS, eIF5B depletion led to a small increase in total eIF2α, while P-eIF2α remained unchanged (Figure S1A,D,E). These results indicate that depletion of eIF5B does not lead to increased phosphorylation of eIF2α and suggest that eIF5B might directly affect expression of ATF4.

To confirm that eIF5B plays a functional role in ATF4 regulation, we assessed the impact of eIF5B depletion on a downstream member of the ATF4 regulon, GADD34. Depletion of eIF5B led to roughly a 3.5- and a 3-fold increase in GADD34 protein levels in HEK293T and U2OS, respectively (Figure 1A,F and Figure S1A,F). RT-qPCR analysis revealed an increase in steady-state levels of the ATF4-encoding mRNA of ~2.2- and 5-fold in HEK293T and U2OS, respectively (Figure 1G and Figure S1G), suggesting that eIF5B might repress transcription and/or promote turnover of the *ATF4* mRNA. Similarly, the mRNA encoding GADD34 increased upon eIF5B depletion by ~3- and 30-fold in HEK293T and U2OS, respectively (Figure 1H and Figure S1H). The increase in steady-state levels of *GADD34* mRNA upon silencing eIF5B was expected, as GADD34 is activated by ATF4 at the level of transcription. Taken together, the data indicate that eIF5B represses ATF4 expression and, consequently, the ATF4-mediated transcriptional regulon.

### 2.2. eIF5B Represses Translation of ATF4

**Figure 2:**
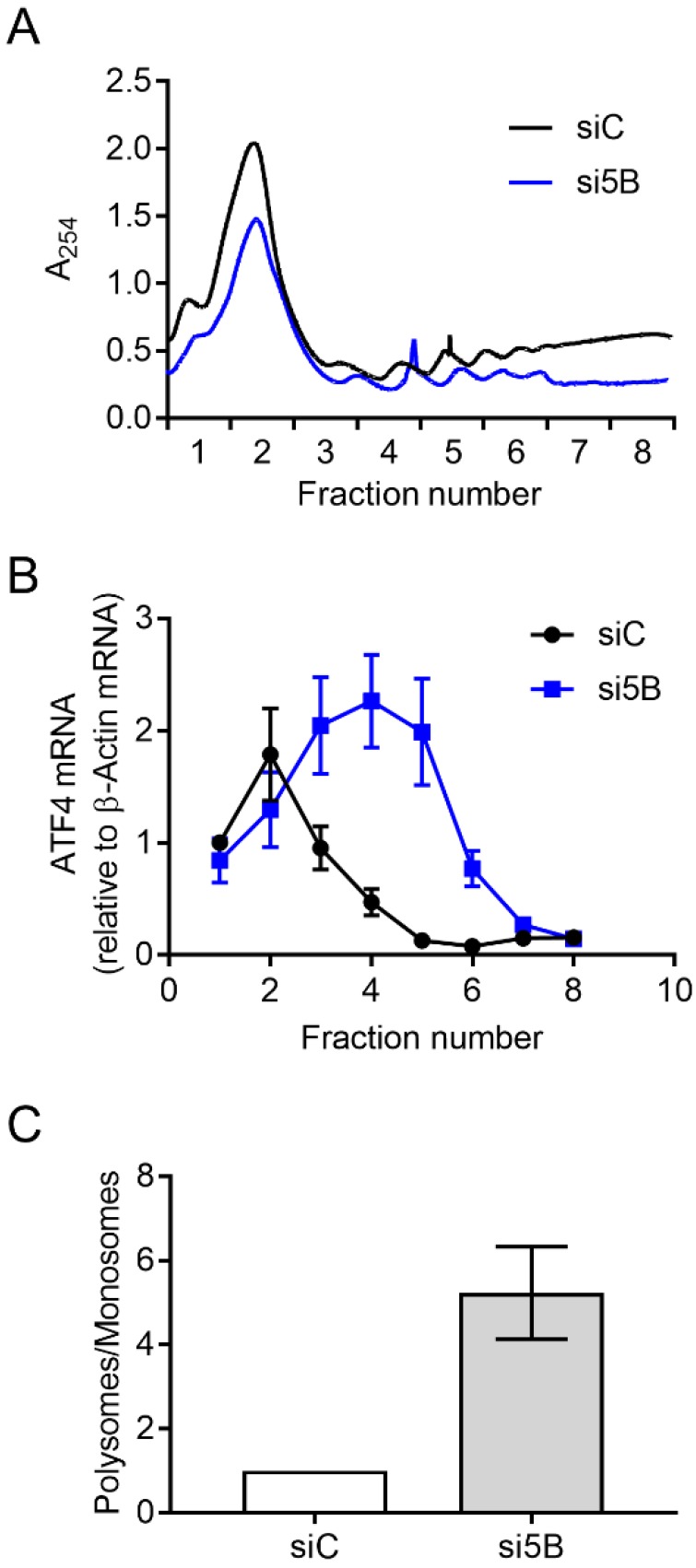
Depletion of eIF5B leads to increased translation of ATF4. HEK293T cells were reverse-transfected with a non-specific control siRNA (siC) or an eIF5B-specific siRNA pool (si5B), incubated 96 h, harvested in RNA lysis buffer, and subjected to polysome profiling analysis. (**A**) A representative polysome profile from control versus eIF5B-depleted HEK293T cells. (**B**) The proportion of *ATF4* mRNA (relative to β-Actin mRNA) for each fraction from the panel (**A**). (**C**) Fractions 1–2 (representing monosomes) were pooled, as were fractions 3–8 (representing polysomes). The ratio of polysomes/monosomes is shown for a representative experiment. Data in panels (**B**,**C**) are expressed as the mean ± SD for technical triplicates. An independent experiment is shown in Figure S2.

Steady-state levels of the ATF4-encoding mRNA increased ~2.3-fold upon eIF5B depletion in HEK293T cells (Figure 1G), lower than the magnitude of the effect on ATF4 protein levels (~3.7-fold; Figure 1C). This suggests that eIF5B might influence ATF4 levels post-transcriptionally. To investigate whether eIF5B represses the translation of *ATF4* mRNA, we conducted polysome profiling to determine the association of *ATF4* mRNA with translating polyribosomes versus monoribosomes. In this assay, cell lysates are fractionated by ultracentrifugation on a sucrose density gradient to separate monosomes from polysomes. Total RNA is isolated from each fraction and RT-qPCR is performed to quantify the association of an mRNA of interest with each fraction. The ratio of mRNA associated with polysomes versus monosomes gives a measure of translation efficiency, independent of steady-state mRNA levels [13,23]. The overall polysome profile of HEK293T cells was not drastically altered by silencing eIF5B (Figure 2A and Figure S2A), indicating a minimal effect of eIF5B depletion on global translation. However, the proportion of *ATF4* mRNA associated with polysomes versus monosomes increased ~5-fold in response to eIF5B depletion (Figure 2B,C), indicating an increased translation of ATF4. This was corroborated by an independent experiment (Figure S2). The observed effect of eIF5B on steady-state levels of the *ATF4* mRNA (Figure 1E) might reflect an indirect effect of eIF5B on transcription or be a consequence of mRNA stabilization due to increased poly-ribosomal transit. Together, the results indicate that eIF5B down-regulates ATF4 at the translational level in HEK293T cells.

### 2.3. eIF5B Facilitates uORF-Mediated Repression of ATF4 Translation

**Figure 3:**
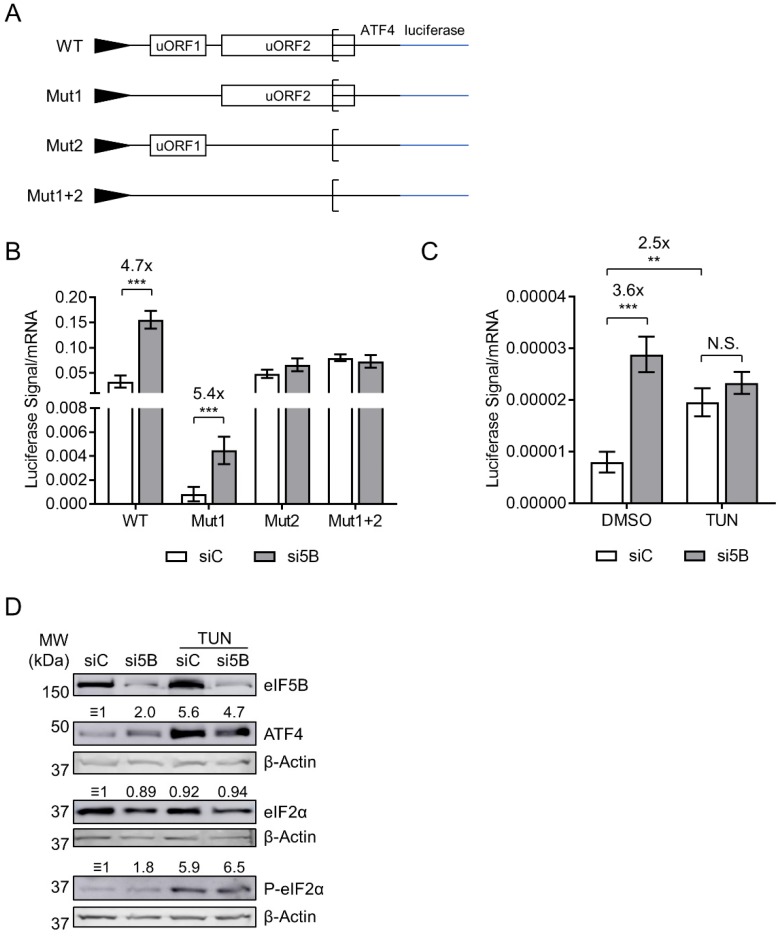
eIF5B represses ATF4 translation by a uORF-dependent mechanism. (**A**) Schematic representation of the ATF4-firefly luciferase reporter fusions used in this study, all transcribed from a minimal TK promoter. Note that Mut1 and Mut2 possess a start codon mutation (ATG to AGG) that inactivates the regulatory functions of uORF1 and uORF2, respectively. Mut1+2 possesses both mutations. (**B**) Control or eIF5B-depleted HEK293T cells were transfected with the above-mentioned plasmids (48 h post siRNA transfection). After another 48 h, the luminescence from firefly luciferase was measured and normalized to the steady-state levels of firefly luciferase mRNA (measured by RT-qPCR). (**C**) Luciferase levels were measured as in panel (**B**), except that the cells were treated with 5 µg/mL tunicamycin (TUN) for the last 6 h of the incubation prior to harvesting. Data are expressed as mean ± SEM for 3 independent replicates. ** *p* < 0.01; *** *p* < 0.001. (**D**) Immunoblots probing for eIF5B, ATF4, eIF2α, P-eIF2α, or β-Actin (internal control). Numbers above the blots represent their quantitation relative to β-Actin.

The initiation of ATF4 translation is controlled by two upstream open reading frames (uORFs) (Figure 3A) [24]. A start codon mutation that inactivates the first (uORF1) was shown to decrease expression of luciferase (mentioned as *ATF4-luc* herein) from an *ATF4*-firefly luciferase fusion mRNA—consistent with a model wherein uORF1 recruits ribosomes onto the mRNA while uORF2, which overlaps the ATF4 ORF, prevents translation initiation of ATF4 [24]. As eIF5B represses the translation of ATF4, we tested whether eIF5B represses expression of the *ATF4-luc* reporter and whether this repression depends on either uORF. Note that these *ATF4-luc* constructs are transcribed from a heterologous promoter [24] and that we normalized the luciferase activity to steady-state levels of *ATF4-luc* mRNA in order to ensure that the results reflected translation of the construct rather than effects on transcription or mRNA turnover. Indeed, depletion of eIF5B led to a ~5-fold increase in translation of wild-type *ATF4-luc* (Figure 3B). Mutation of uORF1 led to an overall decrease in *ATF4-luc* translation, as expected, but depletion of eIF5B still led to a ~5-fold increase (Figure 3B), suggesting that eIF5B is able to repress ATF4 translation when uORF1 is inactive. However, eIF5B depletion had no effect on expression of *ATF4-luc* possessing either mutated uORF2 or the combined uORF1+2 mutations (Figure 3B), suggesting that eIF5B represses ATF4 translation by a mechanism involving the repressive uORF2.

### 2.4. eIF5B Depletion and eIF2α Phosphorylation Do Not Cause a Synergistic Induction of ATF4

The decreased availability of ternary complex under conditions of eIF2α phosphorylation leads to decreased translation initiation of uORF2 and increased initiation at the main ATF4-coding ORF [24]. As expected, treatment with tunicamycin—which blocks N-glycosylation of proteins, resulting in the accumulation of unfolded proteins and thus eIF2α phosphorylation [25]—increased expression of WT *ATF4-luc* (~2.5-fold; Figure 3C). This effect was similar to that of eIF5B depletion (~3.6-fold; Figure 3C). However, eIF5B depletion caused no further increase in *ATF4-luc* translation in tunicamycin-treated cells (Figure 3C). Similar results were obtained when we measured steady-state levels of ATF4 protein (Figure 3D). As expected, tunicamycin treatment lead to an increase in phosphorylation of eIF2α (Figure 3D). The lack of synergy between eIF5B depletion and tunicamycin treatment suggests that they converge on a single point of regulation, such as the delivery of Met-tRNA_i_ to the 40S ribosomal subunit or the availability of ternary complex.

### 2.5. eIF5B Cooperates with eIF1A and eIF5 to Repress ATF4 Translation

**Figure 4:**
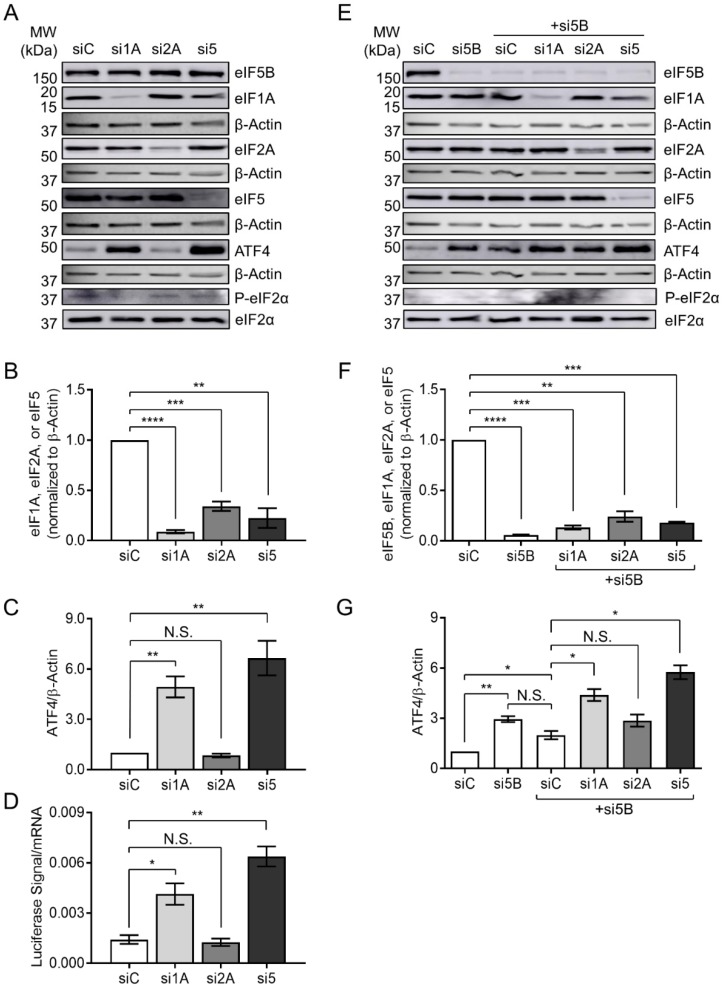
eIF5B cooperates with eIF1A and eIF5, but not eIF2A, to repress ATF4 translation. (**A**) HEK293T cells were transfected with a control siRNA (siC) or siRNAs targeting eIF1A (si1A), eIF2A (si2A), or eIF5 (si5) and subjected to immunoblotting as described for Figure 1. Representative images are shown of immunoblots probing for eIF1A, eIF2A, eIF5, ATF4, eIF2α, P-eIF2α, or β-Actin (internal control). (**B**) Quantitation of eIF1A, eIF2A, or eIF5, each normalized to β-Actin. (**C**) Quantitation of ATF4, normalized to β-Actin. (**D**) HEK293T cells were depleted of eIF1A, eIF2A, or eIF5, as above, before measuring expression of the WT ATF4-luc reporter construct as in Figure 3B. (**E**) HEK293T cells were transfected with a control siRNA (siC), an siRNA targeting eIF5B (si5B), or si5B in combination with siC, si1A, si2A, or si5. Representative immunoblots probing for eIF5B, eIF1A, eIF2A, eIF5, ATF4, eIF2α, P-eIF2α, or β-Actin (internal control), are shown. (**F**) Quantitation of eIF5B, eIF1A, eIF2A, or eIF5, each normalized to β-Actin. (**G**) Quantitation of ATF4, normalized to β-Actin. Data are expressed as mean ± SEM for 3 (panels **B**–**D**) or 2 (panels **F**,**G**) independent biological replicates. * *p* < 0.05; ** *p* < 0.01; *** *p* < 0.001; **** *p* < 0.0001.

eIF1A and eIF5 have recently been shown to compete for binding to eIF5B, suggesting a coordination of their activities during translation initiation [26]. Moreover, eIF2A (an initiator tRNA carrier) has been suggested to function synergistically with eIF5B in the eIF2-independent translation of an IRES-containing mRNA [27]. We, therefore, tested whether any of these eIFs play a role in ATF4 repression. Indeed, depletion of eIF1A and eIF5 led to a ~5- and a 6-fold increase in ATF4 protein levels, respectively, while depletion of eIF2A had no effect (Figure 4A–C). This pattern was matched exactly for expression of the *ATF4-luc* reporter construct (Figure 4D), confirming that eIF1A and eIF5 repress ATF4 post-transcriptionally. In order to test whether eIF5B represses ATF4 expression in coordination with eIF1A, eIF2A, or eIF5, we depleted eIF5B either alone or in combination with each of these factors (Figure 4E,F). Depletion of eIF5B alone or in combination with the non-specific control siRNA led to a ~3-fold and 2-fold increase in ATF4 levels, respectively, while depletion of eIF5B plus eIF2A led to a similar increase (~3-fold), consistent with the lack of eIF2A-mediated regulation (Figure 4E,G). However, depletion of eIF5B in combination with eIF1A or eIF5 led to a ~4-fold and 6-fold increase in ATF4 levels, respectively (Figure 4G). These increases were no larger than those seen upon depletion of eIF1A or eIF5 alone (Figure 4C), suggesting that eIF5B depletion does not synergize with depletion of either eIF1A or eIF5. No increase in total or phospho-eIF2α was apparent upon depletion of any factor, confirming that the observed effects on ATF4 are not due to a general stress-induced phosphorylation of eIF2α. Together, the data suggest that eIF1A and eIF5 cooperate with eIF5B in order to repress ATF4 translation.

## 3. Discussion

In this work, we identify a role for eIF5B in uORF-mediated repression of ATF4 translation initiation. Depletion of eIF5B leads to increased translation of the *ATF4* transcript, and eIF5B-imposed repression of an *ATF4-luciferase* translational reporter fusion requires the repressive uORF2 to be intact (Figure 1, Figure 2 and Figure 3). Although we observed a modest increase in steady-state levels of the *ATF4* mRNA (Figure 1), polysome profiling analysis (Figure 2) and translational reporter assays (Figure 3) demonstrate that eIF5B represses ATF4 expression mainly at the level of translation. Although stress, such as endoplasmic reticulum (ER) stress, leads to transcriptional activation of ATF4 [28], we observed no increase in eIF2α phosphorylation upon eIF5B depletion (Figure 1), suggesting that eIF5B depletion leads to increased levels of *ATF4* mRNA by an alternative mechanism. For instance, the effect of eIF5B on steady-state levels of the *ATF4* mRNA might reflect an indirect effect of eIF5B on transcription (e.g., via regulation of a transcription factor) or be a consequence of mRNA stabilization due to increased ribosomal transit.

Thus far, uORFs have been found in approximately half of human and mouse transcripts, with varied effects on protein expression—typically, uORFs reduce expression by 30–80% [29]. Interestingly, uORFs are common to certain classes of mRNAs. For instance, they are present in two-thirds of oncogenes and in many genes encoding proteins involved in cell differentiation, cell cycle regulation, and the integrated stress response [29]. Reports have shown that ribosomes encountering uORFs either (1) translate the uORF and stall, causing mRNA decay, (2) translate the uORF and, with some probability, reinitiate at the coding sequence, or (3) scan over the uORF [7,9]. uORFs are known to show varying levels of translational regulation based on the nucleotide sequence surrounding the uORF, the distance of the uORF from the CDS, and the number of uORFs present [9]. Importantly, as uORFs can cause a high reduction of protein expression (30–80%), they often affect phenotype. Calvo et al. identified uORFs created or deleted by a polymorphism in 509 genes correlating to at least 24 human diseases, including Alzheimer’s disease, and several tumor types [30]. To date, three rare uORF-altering mutations have been reported to alter levels of essential proteins and cause human diseases: a hereditary form of thrombocythaemia caused by a mutation which eliminates a uORF, a familial predisposition to melanoma caused by the introduction of a uORF, and a hereditary hypotrichosis caused by disruption of a uORF [30,31,32]. Notably, 8–12% of melanoma is linked to mutations in CDKN2A of the chromosome 9 p21 locus, in which an alternative start codon is formed which leads to decreased levels of the functional protein [32]. Thus, understanding the mechanisms by which uORFs regulate gene expression has the potential to affect human phenotype and disease [30].

**Figure 5:**
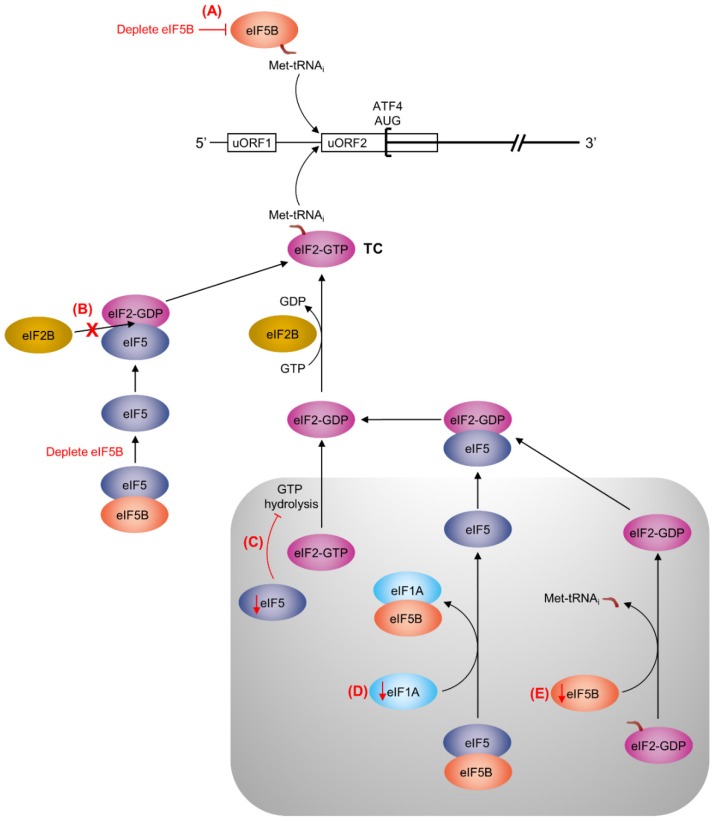
Possible mechanisms for uORF2-mediated repression of ATF4 translation by eIF5B, eIF1A, and eIF5. The *ATF4* mRNA is represented as a horizontal black line, with uORF1 and uORF2 represented as rectangles and the ATF4 start codon represented as a square bracket. In the established mechanism of ATF4 repression, translation re-initiation will tend to occur at the uORF2 start codon when ternary complex (TC) is abundant; however, when TC abundance is decreased (e.g., by phosphorylation of eIF2α), re-initiation will be delayed, allowing the ribosome to bypass uORF2 and initiate translation of ATF4 [24]. Here, we propose several potential mechanisms (**A**–**E**, highlighted in red), for uORF2-mediated repression of ATF4 by eIF5B (light orange), eIF5 (dark blue), and eIF1A (light blue). The pre-initiation complex (PIC) is represented by the grey box. Red arrows indicate a decrease of eIF5, eIF1A, or eIF5B. Note that this is not meant to be an exhaustive list and that any one (or any combination) of these mechanisms might be at play. (**A**) eIF5B might deliver Met-tRNA_i_ to uORF2, providing an alternative to eIF2-GTP (pink). Depletion of eIF5B would consequently decrease translation initiation at the uORF2 start codon, increasing the translation of ATF4. (**B**) As eIF5B interacts with eIF5 [26], depletion of eIF5B could lead to an increase in free eIF5. Available eIF5 can form a complex with eIF2-GDP that can prevent the interaction of eIF2 with eIF2B (yellow), slowing TC re-formation [33,34]. (**C**) Depletion of eIF5 would inhibit GTP hydrolysis by eIF2 [33,35,36,37], preventing its release from the PIC and, subsequently, TC re-formation. (**D**) Depletion of eIF1A would prevent the displacement of eIF5B from eIF5 [26], inhibiting the release of eIF5 and eIF2 from the PIC and, thus, inhibiting TC re-formation. (**E**) Depletion of eIF5B would inhibit the displacement of eIF2-GDP from Met-tRNA_i_ [33,35,36,37], thus inhibiting TC re-formation.

We show in this work that repression of ATF4 translation by eIF5B is unaffected by mutation of uORF1, but requires uORF2 to be intact (Figure 3B). The existing literature indicates that uORF1 promotes ATF4 translation, as disruption of uORF1 causes decreased expression of *ATF4-luc* [24]. Moreover, translation of this uORF1 mutant still increases in the presence of thapsigargin, indicating that upregulation of ATF4 translation under conditions of eIF2α phosphorylation does not depend on this uORF [24]. Conversely, disruption of uORF2 causes increased expression of *ATF4-luc*, which becomes insensitive to thapsigargin, indicating that uORF2 is responsible for inhibiting ATF4 translation initiation when the ternary complex is abundant [24]. Thus, eIF5B appears to play a role in facilitating translation initiation at uORF2 instead of the main ATF4-coding ORF, similar to the situation when the ternary complex is abundant. We also observed increased expression of the WT *ATF4-luc* construct upon treating the cells with tunicamycin (Figure 3C). Similar to thapsigargin, tunicamycin leads to phosphorylation of eIF2α [25] and thus limits ternary complex re-formation. Strikingly, the effects of tunicamycin treatment and eIF5B depletion were not additive (Figure 3C), suggesting that both eIF5B and eIF2 converge on a single point of regulation, such as the delivery of Met-tRNA_i_ during translation initiation. If eIF5B is capable of delivering Met-tRNA_i_ to uORF2, then depletion of eIF5B might decrease the probability of translation initiation at uORF2 and increase the probability of initiation at the ATF4 main coding ORF, similar to the situation when eIF2α is phosphorylated (Figure 5A). This could explain why no additive increase in ATF4 translation was observed when eIF5B depletion was combined with tunicamycin treatment.

Notably, an alternatively spliced variant of the human ATF4 mRNA can be translated from an IRES [38]. However, this does not represent the majority of human ATF4 transcripts. Moreover, the *ATF4-luc* reporter used in this work does not represent the IRES-encoding splice variant [24]. While it is possible that eIF5B regulates the IRES element present in the ATF4 splice variant, we observe an increase in ATF4 translation upon eIF5B depletion (Figure 1, Figure 2 and Figure 3), which is in direct opposition to the established role of eIF5B in positively regulating IRES-dependent translation [13,17,18,19]. Moreover, no change (in either direction) was observed upon eIF5B depletion when uORF2 was mutated (Figure 3B), confirming that eIF5B-mediated repression of ATF4 involves this uORF. Together, these observations suggest that eIF5B represses translation of human ATF4 by a uORF- rather than IRES-mediated mechanism, although we cannot rule out the possibility that eIF5B plays an additional role in IRES-mediated translation in the case of the splice variant.

Recent work has shown that eIF2A might function in a complex with eIF5B for the eIF2-independent translation of an IRES-encoding mRNA [27]. In this model, eIF2A functions as the Met-tRNA_i_ carrier while eIF5B provides GTP-, mRNA- and ribosome-binding functions [27]. However, depletion of eIF2A had no effect on ATF4 levels (Figure 4A–D). Moreover, the combined depletion of eIF2A and eIF5B had no effect on ATF4 levels above the effect of eIF5B depletion alone (Figure 4E–G). These data suggest that eIF2A plays no role in the repression of ATF4 by eIF5B.

In contrast, depletion of eIF1A led to a robust increase in ATF4 translation, as did eIF5-depletion (Figure 4A–D). Moreover, depletion of eIF1A in combination with eIF5B led to no further increase than depletion of eIF1A alone, and depletion of eIF5 plus eIF5B led to no further increase than depletion of eIF5 alone (Figure 4C,G), suggesting that eIF5B cooperates with these factors to repress ATF4 translation. An interaction between eIF1A and eIF5B is known to promote translation [39]. In fact, eIF5B overexpression has been shown to suppress the effects of a mutation in eIF1A [40], suggesting a certain amount of functional redundancy. Recent work shows that eIF1A and eIF5 compete for binding to eIF5B in the context of a pre-initiation complex (PIC) in canonical translation initiation [26]. eIF5 is the GTPase-activating protein that promotes GTP hydrolysis by eIF2 upon delivery of Met-tRNA_i_ to the start codon, at which point eIF2 is displaced from Met-tRNA_i_ by eIF5B-GTP and is released as an eIF2:eIF5 complex [33,35,36,37]. Upon ribosomal subunit joining, eIF5B hydrolyzes GTP and is released along with eIF1A [41,42,43,44]. Lin et al. suggest a mechanism for coordination between the steps of start codon selection and ribosomal subunit joining: displacement of eIF2 from Met-tRNA_i_ by eIF5B upon subunit joining may be coupled to the eIF1A-mediated displacement of eIF5 from eIF5B, enabling the eIF2-GDP:eIF5 complex to leave the ribosome [26].

In *Saccharomyces cerevisiae*, overexpression of eIF5 mimics the effect of eIF2α phosphorylation, promoting translation of the yeast equivalent of the ATF4 protein, GCN4 [33]. Specifically, overexpression of eIF5 in yeast increases the levels of an eIF2-eIF5 complex, which prevents eIF2B interaction and thus ternary complex re-formation [33]. Similarly, in human cells, overexpression of eIF5 or its mimic (eIF5 mimicking protein) perturbs the function of eIF2 and induces ATF4 translation by delaying re-initiation at uORF2 [34]. As eIF5B interacts with eIF5 [26], it is possible that depletion of eIF5B leads to an increase in available eIF5, which would bind eIF2 and prevent ternary complex formation, leading to increased translation of ATF4 (Figure 5B). Similarly, eIF5 depletion would prevent GTP hydrolysis by eIF2, slowing its release from the PIC and subsequent re-formation of the ternary complex (Figure 5C). eIF1A depletion would prevent the displacement of eIF5 from eIF5B, slowing the release of eIF5:eIF2-GDP and subsequent reformation of the ternary complex (Figure 5D). Finally, depletion of eIF5B itself could slow ternary complex re-formation by preventing the displacement of eIF2-GDP from PIC-bound Met-tRNA_i_ (Figure 5E). Altogether, we suggest that any perturbation of the stoichiometry of eIF1A, eIF5, and/or eIF5B might lead to decreased translation of uORF2 and, thus, de-repression of ATF4 translation.

Overall, this work demonstrates a role for eIF5B in the uORF2-mediated repression of ATF4 translation—a role which also involves eIF1A and eIF5. Given the prevalence of uORFs in human transcripts, we suggest that eukaryotic initiation factors like eIF5B, eIF1A, and eIF5 might influence the translation of a previously unappreciated number of non-canonically translated mRNAs.

## 4. Materials and Methods

### 4.1. Cell Culture and Reagents

All cell lines were propagated in Dulbecco’s high modified Eagle’s medium (DMEM; HyClone, Logan, UT, USA) with 4 mM l-glutamine, 4500 mg/L glucose, and 1 mM sodium pyruvate, supplemented with 10% fetal bovine serum (FBS; Gibco, Waltham, MA, USA) and 1% penicillin-streptomycin (Gibco). Cells were incubated at 37 °C in a humidified 5% CO2 incubator. Cell lines were routinely tested for mycoplasma contamination with a PCR mycoplasma detection kit (Applied Biological Materials, Richmond, BC, Canada). Reverse transfections were carried out using Lipofectamine RNAiMAX (Invitrogen, Carlsbad, CA, USA) according to the manufacturer’s instructions. Non-specific control siRNA (siC) was obtained from Qiagen (Venlo, Netherlands). Stealth RNAi^TM^ siRNAs targeting eIF5B (HSS114469/70/71) were obtained from Invitrogen. siRNA smart pools targeting eIF1A (M-011262-02-0005), eIF2A (M-014766-01-0005), and eIF5 (M-021336-00-0005) were obtained from Dharmacon (Lafayette, LA, USA). Tunicamycin was obtained from Sigma-Aldrich (St. Louis, MO, USA).

### 4.2. Western Blotting

HEK293T cells were seeded at 200,000 cells/well and reverse-transfected in 6-well plates. After 96 h of incubation, cells were harvested in RIPA lysis buffer supplemented with protease and phosphatase inhibitors. Equal amounts of soluble protein (typically 25 µg per well) were resolved by SDS-PAGE and transferred onto nitrocellulose membranes (GE healthcare, Chicago, IL, USA). Individual proteins were detected by immunoblotting with the antibodies described in Table S1. Primary antibodies were detected with anti-rabbit-HRP conjugate (Abcam, Cambridge, UK) in an AI600 imager (GE healthcare) and densitometry performed using the AI600 analysis software.

### 4.3. Polysome Profiling

HEK293T cells were seeded at 1 million cells per plate and reverse transfected in two 10-cm Petri plates per condition. After 96 h, the control or eIF5B-depleted cells were pooled, lysed in RNA lysis buffer, and subjected to polysome profiling as previously described [13,23]. Gradients were fractionated using a BR-188 density gradient fractionation system (BRANDEL). In the experiment presented in Figure 2, the levels of ATF4 mRNA are normalized to β-Actin mRNA. In the experiment shown in Figure S2, the fractions were spiked with 100 ng of an in vitro transcribed chloramphenicol acetyltransferase (CAT) RNA, to ensure technical consistency in RNA isolation [13]. RNA was isolated essentially as described [23] except that proteinase K treatment was replaced by incubation with 1% SDS at 65 °C for 1 min, and hot acid phenol:chloroform (5:1; Ambion, Waltham, MA, USA) was used to extract the RNA for RT-qPCR analysis.

### 4.4. Luciferase Reporter Assays

HEK293T cells were seeded at 8000 cells per well in a 96-well plate and reverse transfected with control or eIF5B-specific siRNAs. After 48 h, the cells were forward transfected with plasmids encoding the following *ATF4-luc* reporters: WT (p759), Mut1 (p760), Mut2 (p761), and Mut1+2 (p762). The ATF4-luciferase reporters were a kind gift from Dr. Ronald Wek [24]. After a further 48 h, the cells were lysed and luciferase activity measured using a firefly luciferase assay kit (E1500; Promega (Madison, WI, USA)) and a Cytation 5 plate reader (BioTek, Winooski, VT, USA). Immediately following the readings, RNA (for RT-qPCR analysis) was extracted from the lysates as described above.

### 4.5. RT-qPCR

After ethanol precipitating the RNA, cDNA was generated from equal volumes of RNA using the qScript cDNA synthesis kit (Quanta Biosciences, Beverly, MA, USA). Quantitative PCR was performed in a CFX-96 real-time thermocycler (Bio-Rad, Hercules, CA, USA) with PerfeCTa SYBR Green SuperMix (Quanta Biosciences) according to manufacturer’s instructions. Primers are detailed in Table S1. Negative controls without template DNA were run in triplicate. Each reaction was run in triplicate with the following cycle conditions: 1 cycle at 95 °C for 3 min followed by 45 cycles of 95 °C for 15 s, the annealing temperature indicated in Table S1 for 35 s, and 72 °C for 1 min. A melting curve step was added to check the purity of the PCR product. This step consisted of a ramp of the temperature from 65 to 95 °C at an increment of 0.5°C and a hold for 5 s at each step. Expression levels of *ATF4* and *GADD34* mRNAs (relative to β-Actin mRNA) were determined using the ∆Ct method. All other expression levels were determined by the standard curve method.

### 4.6. Statistical Analyses

Unless otherwise specified, all quantitative data represent the mean ± standard error on the mean (SEM) for at least 3 independent biological replicates. Statistical significance was determined by an unpaired, two-tailed t-test without assuming equal variance. The significance level was set at a p-value of 0.05. Data were analyzed using GraphPad Prism, version 7 (GraphPad Software, San Diego, CA, USA).

## Supplementary Materials

Supplementary materials can be found at http://www.mdpi.com/1422-0067/19/12/4032/s1.
