# Supplementary material for: Eukaryotic Initiation Factor 5B (eIF5B) Cooperates with eIF1A and eIF5 to Facilitate uORF2-Mediated Repression of ATF4 Translation
Source: Int J Mol Sci. 2018 Dec 13;19(12):4032. doi: 10.3390/ijms19124032 (PMC6321046; doi:10.3390/ijms19124032)
Supplement: Supplementary file 1 [file ijms-19-04032-s001.pdf]

Table S1. qPCR primers and antibodies used in this study.

Target	Primer	Sequence (5' to 3') or catalogue #	Annealing Temp (°C)	Reference or source
ATF4	-	QuantiTect primer assay, QT00074466	55	Qiagen
GADD34	-	QuantiTect primer assay, QT00013321	55	
Firefly Luciferase	Fw<br>Rv	TCGAAATGTCCGTTCGGTTG<br>CGCAACTGCAACTCCGATAA	50.6	This study
β-actin	Fw<br>Rv	TCACCCACACTGTGCCCATCTACGA<br>TGAGGTAGTCAGTCAGGTCCC	55	[1]
CAT	Fw<br>Rv	GCGTGTTACGGTGAAAACCT<br>GGGCGAAGAAGTTGTCCATA	52	[2]

Company	Antibody Target	Catalogue number
Abcam	Secondary: Goat anti-rabbit-HRP conjugate	ab97051
ProteinTech	eIF5B	13527-1-AP
	eIF2A	11233-1-AP
	eIF5	11155-1-AP
Bio-Rad	β-actin (hFAB Rhodamine)	12004163
Abcam	GADD34	Ab126075
	eIF1A	Ab177939
Cell Signalling Tech.	ATF4	11815
	eIF2α	9722
	Phospho eIF2α	9721

1. Khan, D.; Katoch, A.; Das, A.; Sharathchandra, A.; Lal, R.; Roy, P.; Das, S.; Chattopadhyay, S.; Das, S., Reversible induction of translational isoforms of p53 in glucose deprivation. *Cell death and differentiation* **2015,** 22, (7), 1203-18.
2. Thakor, N.; Holcik, M., IRES-mediated translation of cellular messenger RNA operates in eIF2alpha- independent manner during stress. *Nucleic acids research* **2012,** 40, (2), 541-52.

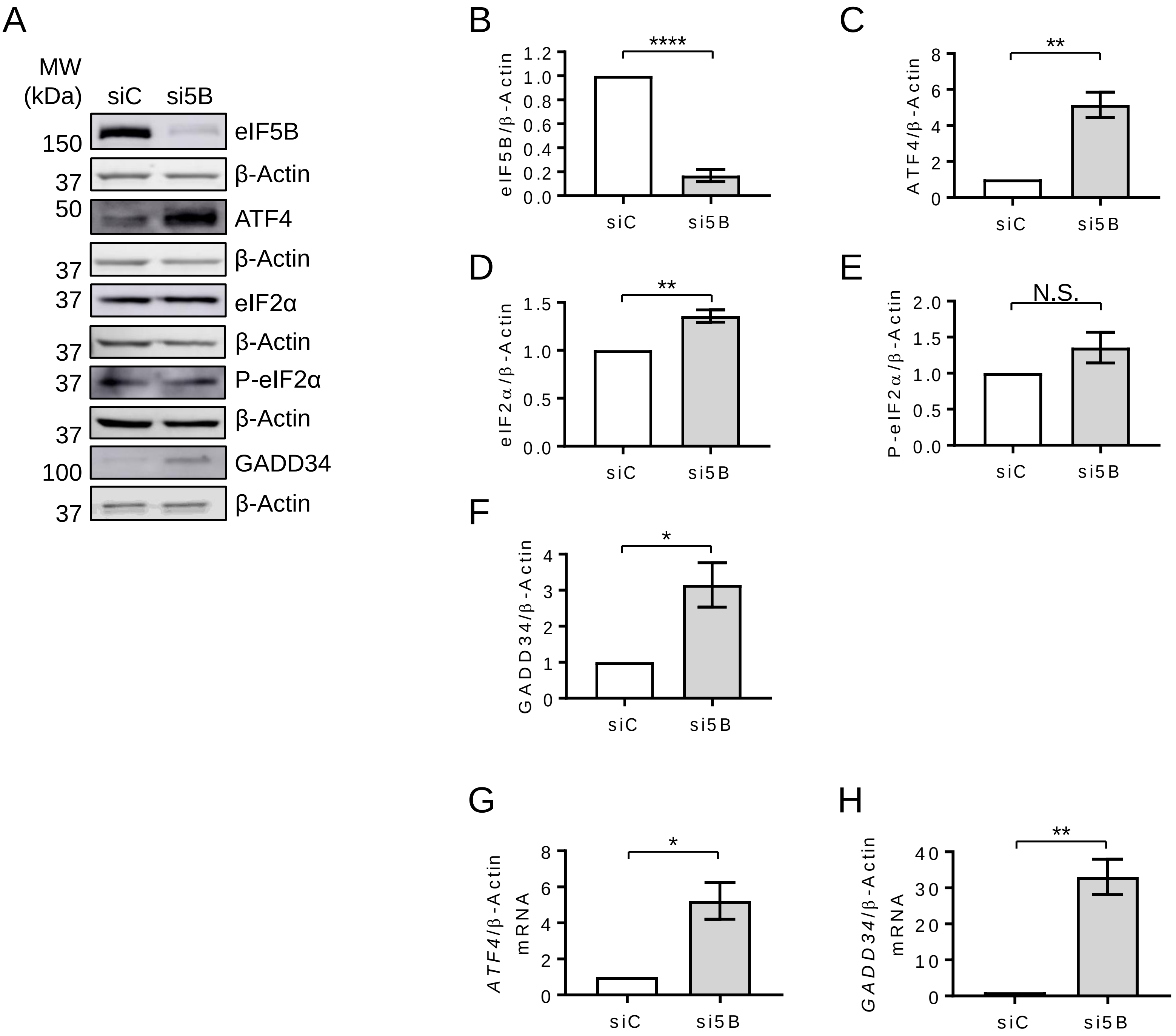

Figure S1. Depletion of eIF5B leads to increased levels of the ATF4 protein in U2OS cells. U2OS were reverse-transfected with a non-specific control siRNA (siC) or an eIF5B-specific siRNA pool (si5B), incubated 96 hours, harvested in RIPA lysis buffer, and 20 µg of total protein resolved by SDS-PAGE before performing immunoblotting. **(A)** Representative images of immunoblots probing for eIF5B, ATF4, eIF2α, P-eIF2α, GADD34, or β-actin (internal control). **(B-F)** Quantitation of eIF5B **(B)**, ATF4 **(C)**, eIF2α **(D)**, P-eIF2α **(E)**, or GADD34 **(F)**, normalized to β-actin, from U2OS cells. **(G, H)** Total RNA was isolated from control or eIF5B-depleted U2OS cells and subjected to RT-qPCR analysis of steady-state mRNA levels for *ATF4* **(G)** or *GADD34* **(H)**, normalized to β-actin mRNA. Data are expressed as mean ± SEM for at least 3 (B-E, G, H) and up to 4 (F) independent biological replicates. *, $p < 0.05$; **, $p < 0.01$; ***, $P < 0.001$; ****, $p < 0.0001$.

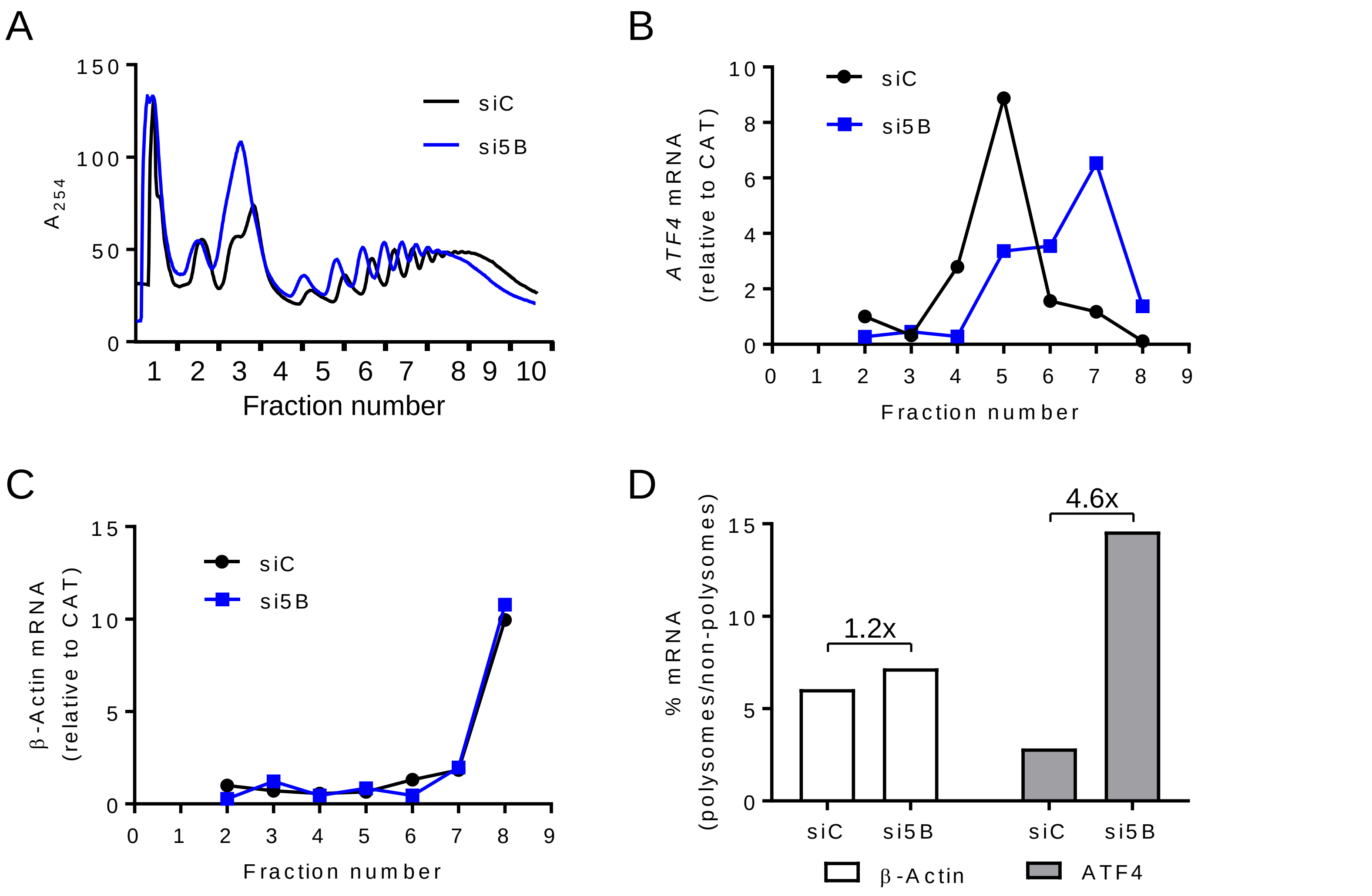

Figure S2. Depletion of eIF5B increases translation of ATF4 in HEK293T cells (related to Figure 2). Control and eIF5B-depleted HEK293T cells were subjected to polysome profiling analysis as described in Materials and Methods. **(A)** Polysome profile from control versus eIF5B-depleted cells. **(B)** The proportion of *ATF4* mRNA for each fraction from panel (A) was normalized to an external control RNA (chloramphenicol acetyltransferase, CAT). **(C)** The proportion of β-actin mRNA for each fraction from panel (A) was normalized to CAT. **(D)** Fractions 1-3 (representing monosomes) were pooled, as were fractions 4-10 (representing polysomes). The ratio of polysomes/monosomes is shown.
Note that this experiment was conducted by N. Thakor at the Children's Hospital of Eastern Ontario (CHEO) in 2012 and thus constitutes a totally independent replicate from that shown in Figure 2.
